# Appearance-related attentional bias is associated with dysmorphic appearance concern in individuals with jaw deformity: an eye-tracking study

**DOI:** 10.3389/fpsyt.2026.1739028

**Published:** 2026-04-23

**Authors:** Kiki Matsuzawa, Motoyasu Honma, Shugo Haga, Sumire Ogura, Yuri Masaoka, Masahiko Izumizaki, Haruhisa Nakano

**Affiliations:** 1 Department of Orthodontics, Showa Medical University School of Dentistry, Tokyo, Japan; 2 Department of Physiology, Showa Medical University School of Medicine, Tokyo, Japan

**Keywords:** attentional bias, body dysmorphic disorder, body image concern inventory, eye-tracking, jaw deformity

## Abstract

**Introduction:**

Appearance-related attentional bias has been observed in body dysmorphic disorder (BDD) and in individuals with elevated appearance concern; however, it remains unclear whether similar attentional biases are observed in individuals with objectively verifiable jaw deformities. Appearance-related attentional bias has been observed across clinical populations with elevated appearance concern, highlighting its relevance as a general perceptual-cognitive mechanism associated with appearance monitoring.

**Methods:**

Sixty patients with jaw deformities and 40 age-matched controls completed the Body Image Concern Inventory (BICI), a validated measure of dysmorphic appearance concern, and underwent eye-tracking while viewing photographs of their own and others’ faces. Gaze duration was analyzed across six facial regions (forehead, eyes, nose, mouth, jaw, and others), and associations between gaze patterns and BICI scores were examined.

**Results:**

Patients exhibited significantly higher BICI scores than controls. During both self- and other-face viewing, patients showed prolonged gaze duration toward the jaw region. Furthermore, exploratory correlation analyses showed that gaze duration was positively associated with BICI scores within the patient group during viewing of both self and others’ faces.

**Discussion:**

Individuals with jaw deformities exhibit elevated dysmorphic appearance concern and increased attention to perceptually salient facial features. These findings characterize appearance-related attentional biases in individuals with jaw deformities and provide insight into perceptual processes associated with dysmorphic appearance concern.

## Introduction

Body dysmorphic disorder (BDD) is a psychiatric condition defined by a preoccupation with perceived flaws in appearance, often involving exaggerated attention to minor or imagined defects (1–3). Eye-tracking studies have shown that individuals with BDD allocate disproportionate attention to disliked features while avoiding socially salient cues such as the eyes (4, 5). However, little is known about how these mechanisms manifest in individuals with objectively visible deformities, where appearance-related concerns intersect with real morphological differences. Importantly, emerging evidence suggests that such appearance-related attentional biases in BDD may not be strictly confined to self-face perception but may also influence the processing of others’ faces, reflecting generalized self–other attentional patterns (4–6). However, whether similar attentional patterns emerge in individuals with objectively verifiable facial deformities remains unclear. In the present study, we examined dysmorphic appearance concern as a psychological dimension and its relationship with visual attentional patterns in individuals with jaw deformities.

Facial deformities, such as jaw deformity requiring orthognathic surgery, profoundly influence self-perception and psychosocial well-being (7–9). Although surgical correction often improves facial aesthetics and quality of life, a subset of patients continues to experience dissatisfaction and distress despite objective improvement (10, 11). These discrepancies suggest that perceptual-cognitive factors, rather than physical deformity alone, may drive persistent psychological impairment. Indeed, recent evidence indicates that approximately 10% of orthognathic candidates exhibit clinically relevant dysmorphic appearance concern (12), highlighting the importance of appearance-related psychological processes associated with perception and self-monitoring in this population.

Attentional bias provides a promising behavioral index for investigating these perceptual-cognitive mechanisms. Appearance-related attentional bias, characterized by increased attention to perceived appearance-relevant features, may sustain negative self-appraisal and influence face processing (6). Aberrant allocation of visual attention may therefore reflect dysfunctional self-monitoring loops common across appearance-related psychopathologies.

The present study aimed to quantify gaze behavior and dysmorphic appearance concern in individuals with jaw deformities. Specifically, we hypothesized that patients would report higher levels of appearance-related concern than healthy controls, and they would exhibit attentional bias—manifested as prolonged gaze on the jaw region during face viewing. Furthermore, we examined whether attentional bias extends beyond self-face perception and influences attention to others’ faces.

## Methods

### Participant

This was a cross-sectional observational study comparing patients with jaw deformities and age-matched healthy controls. We used the software (G*Power 3.1) to determine the required sample size. The assumed medium effect size (f = 0.25) was based on prior eye-tracking studies in BDD and related populations, which typically reported medium-sized group differences in gaze duration and attentional allocation with sample sizes ranging from approximately 40 participants per group (e.g., 4–6). The patient group comprised 60 individuals who underwent orthognathic surgery, including bilateral sagittal split osteotomy with or without Le Fort I osteotomy (mean age 26.5 years; range 18–44; 40 females) (13, 14). Exclusion criteria included age ≥ 50 years, comorbid conditions other than jaw deformity, and surgical procedures outside the specified scope. A control group of 40 age-matched healthy individuals was also recruited (mean age 26.6 years; range 20–37; 27 females). An unpaired t-test confirmed no significant age differences between groups (*p* = 0.985). None of the participants reported a history of psychiatric or neurological disorders. No formal psychiatric diagnostic interviews were conducted; psychiatric history was assessed based on self-report. Therefore, no psychiatric diagnosis, including Body Dysmorphic Disorder, was made or inferred in the present study. To minimize selection bias, all eligible surgical candidates during the study period were invited to participate. No participants were excluded, and none withdrew from the study.

### Procedures

All experiments, including psychological assessments and eye-tracking sessions, were carried out in a controlled laboratory setting. The primary outcome was dysmorphic appearance concern, measured using the Body Image Concern Inventory (BICI) (15). The main exposure variable was the presence of jaw deformity (patient vs. control). Secondary variables included eye-tracking indices (total gaze duration and gaze count on predefined facial regions of interest: forehead, eyes, nose, mouth, jaw, and outer). Potential confounders, such as age, were controlled through group matching. Experimenters were blinded to participants’ group identity during eye-tracking data analysis. To reduce reporting bias, psychological questionnaires were completed anonymously, and participants were instructed not to discuss their condition during testing.

### Clinical assessment

Dysmorphic appearance concern was assessed using the BICI, a validated 19-item self-report questionnaire measuring appearance-related concern and associated cognitive and behavioral responses. The BICI is widely used in psychological research to assess dysmorphic appearance concern as a dimensional construct and enables examination of individual differences in appearance-related concern. The total score was used for analyses. The validity of the Japanese version of the BICI has been established (16).

### Gaze measurement

**Figure 1:**
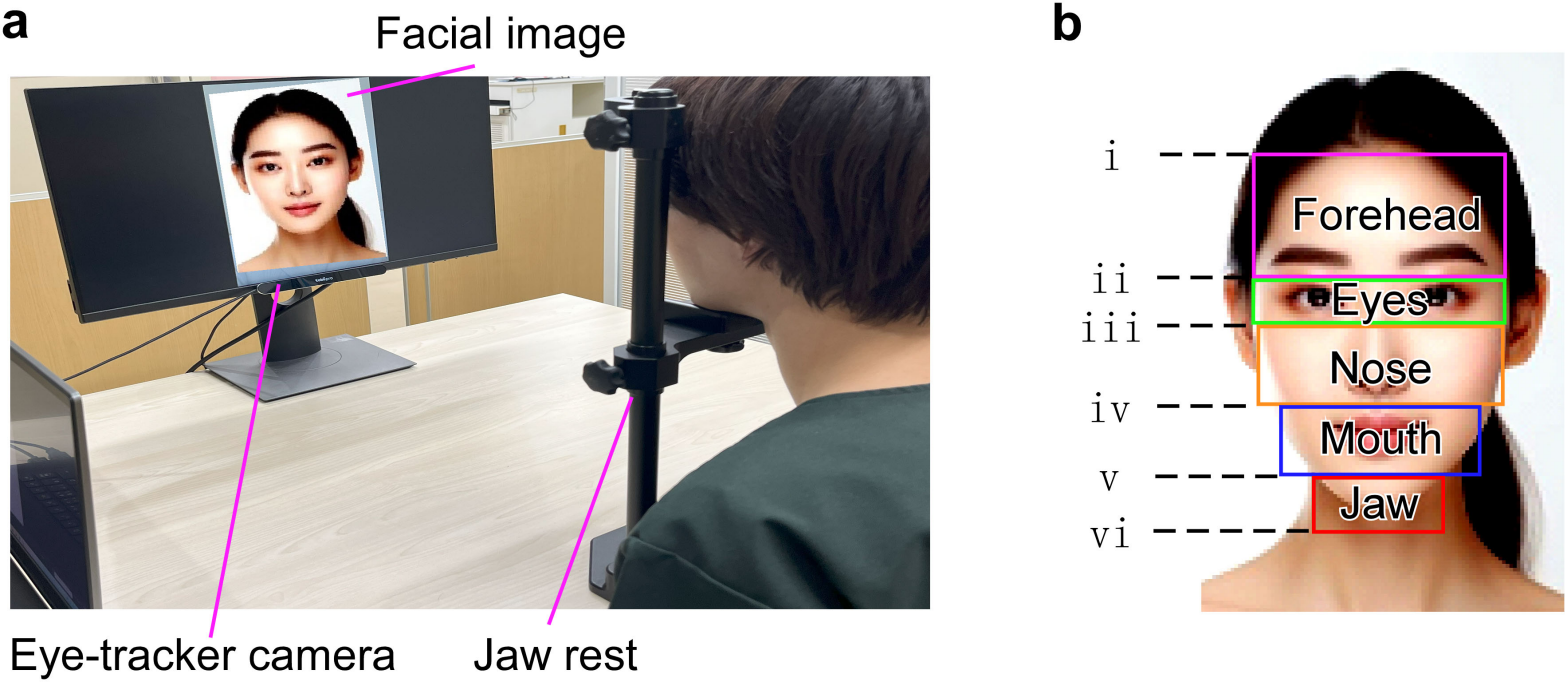
Experimental setup and gaze pattern analysis. **(a)** Experimental environment. Participants stabilized their head using a jaw rest and forehead pad and fixated on facial images presented on a PC monitor. Eye movements were recorded using an infrared-based eye-tracking device positioned below the monitor. **(b)** Define of regions for gaze pattern analysis. Gaze duration was calculated across six regions of interest (forehead, eyes, nose, mouth, jaw, and outer facial region). Lines i–vi indicate the vertical boundaries used to define these regions. The forehead region was defined vertically from the hairline (i) to the midpoint between the eyebrows and eyes (ii). The eyes region extended from (ii) to a line below the eyes (iii), corresponding to the same vertical distance as (ii). The nose region was defined from (iii) to the area between the lower nose and the mouth (iv). The mouth region extended from (iv) to the area between the mouth and the lower jaw (v). The jaw region spanned from (v) to a point (vi), determined by projecting the distance between (v) and the lower jaw downward. For all five regions, the horizontal boundaries were set as rectangles encompassing the facial skin. The remaining area outside these five regions was defined as the outer region. Source: Facial images are fictional images generated by the authors. No likeness rights apply.

The gaze trajectories of participants were measured when they were shown photographs of their self-faces and of stranger-faces (**Figure 1a**). The experimenter asked the participants to observe the facial image. Three types of facial photographs were prepared, including frontal and left/right profile views, taking into account the factor of face angle. The photographs of the participants’ self-faces were taken using a digital camera with a 3D facial scanning device (RFS200 face scanner, Ray Co., Ltd.) immediately before the test. To take into account the factor of novelty, the stranger-faces were unknown faces that the person had never seen before. Four stranger-faces (two women and two men) were prepared. The eye-tracking device was the Eyes Tribe Tracker sensor (Tobii Technology KK, Stockholm, Sweden). The sampling rate was 30 Hz, because the primary outcome of the present study was gaze duration rather than fine-grained saccade dynamics. The data from the dominant eyes was used for analysis. The effectiveness was assessed by identifying the eye for which closing one eye did not alter the visual field compared with viewing with both eyes. The participants sat in chairs and their jaws were fixed in place with jaw rests. The distance from the participants’ eyes to the screen was 47.5 cm. The facial photograph size was 300 mm in length and 195 mm in width (visual angle: 35.1 degree in length and 23.4 degree in width). The brightness in the laboratory was approximately 5,000 lux. After the written consent and clinical assessment, each participant observed 15 facial photographs (3 angles; frontal and left/right profiles x 5 faces; one self and 4 strangers). The presentation order of 15 photographs was randomized. Each facial photograph was presented for 15 seconds with 15-second breaks, and the participants’ eyes movement was analyzed during this time. To quantitatively assess gaze distribution, each face was divided into six regions of interest: forehead, eyes, nose, mouth, jaw, and outer region (**Figure 1b**).

### Statistical analysis

No arbitrary grouping or median splits were applied to preserve data variability. The unpaired t-test was performed for clinical assessment. A three-way analysis of variance (ANOVA) was performed on gaze data; region (forehead, eyes, nose, mouth, jaw, and outer), group (patient and control); face identity (self and stranger). For all repeated-measures ANOVAs, the assumption of sphericity was tested using Mauchly’s test. In cases where sphericity was violated, Greenhouse–Geisser–corrected degrees of freedom and *p*-values were reported. Data on the three faces of frontal, left, and right profiles were combined into one sample on each region in the case of self-face. In the case of stranger-face, the three profiles on all the 4 persons were combined into one sample on each region. *Post hoc* test with Bonferroni correction was conducted to compare group differences on each region. Pearson’s correlation was performed to test relationships between the clinical assessment score and gaze duration. In this correlation analysis, results were calculated without correction. All tests were two-tailed. The results are presented as mean ± standard error of the mean and effect sizes (*η*^2^). Statistical significance was set at *p* value < 0.05. SPSS version 31 for Windows (IBM, Inc., Chicago, IL) was used for statistical analyses.

## Results

### Clinical assessment

**Figure 2:**
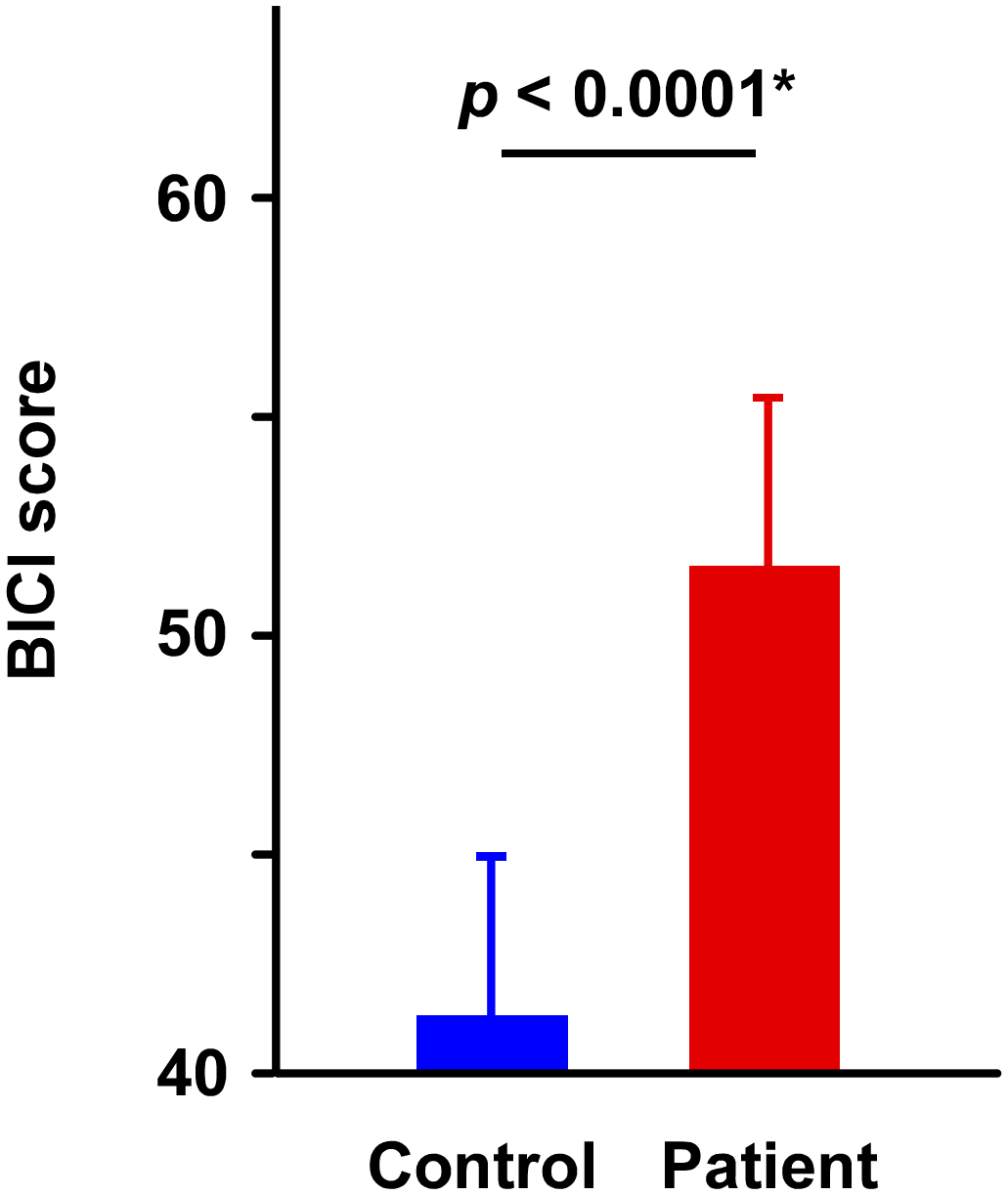
The result of BICI score. Patients scored significantly higher than healthy controls. Asterisks indicate significant differences (*p < 0.0001). Error bars represent the standard error of the mean (SEM).

In the clinical assessment of BICI, the Levene test showed a significant difference between groups (*F*_(1, 98)_ = 10.297, *p* = 0.002), and then an unpaired Welch’s t-test revealed that the score in the patient group was higher than that in the healthy group (*t*_97.73_ = 4.384, *p* < 0.0001, Cohen’s *d* = 0.835). The group comparison indicates that the patient group tended to show higher levels of appearance-related concern (**Figure 2**).

### Gaze measurement

**Figure 3:**
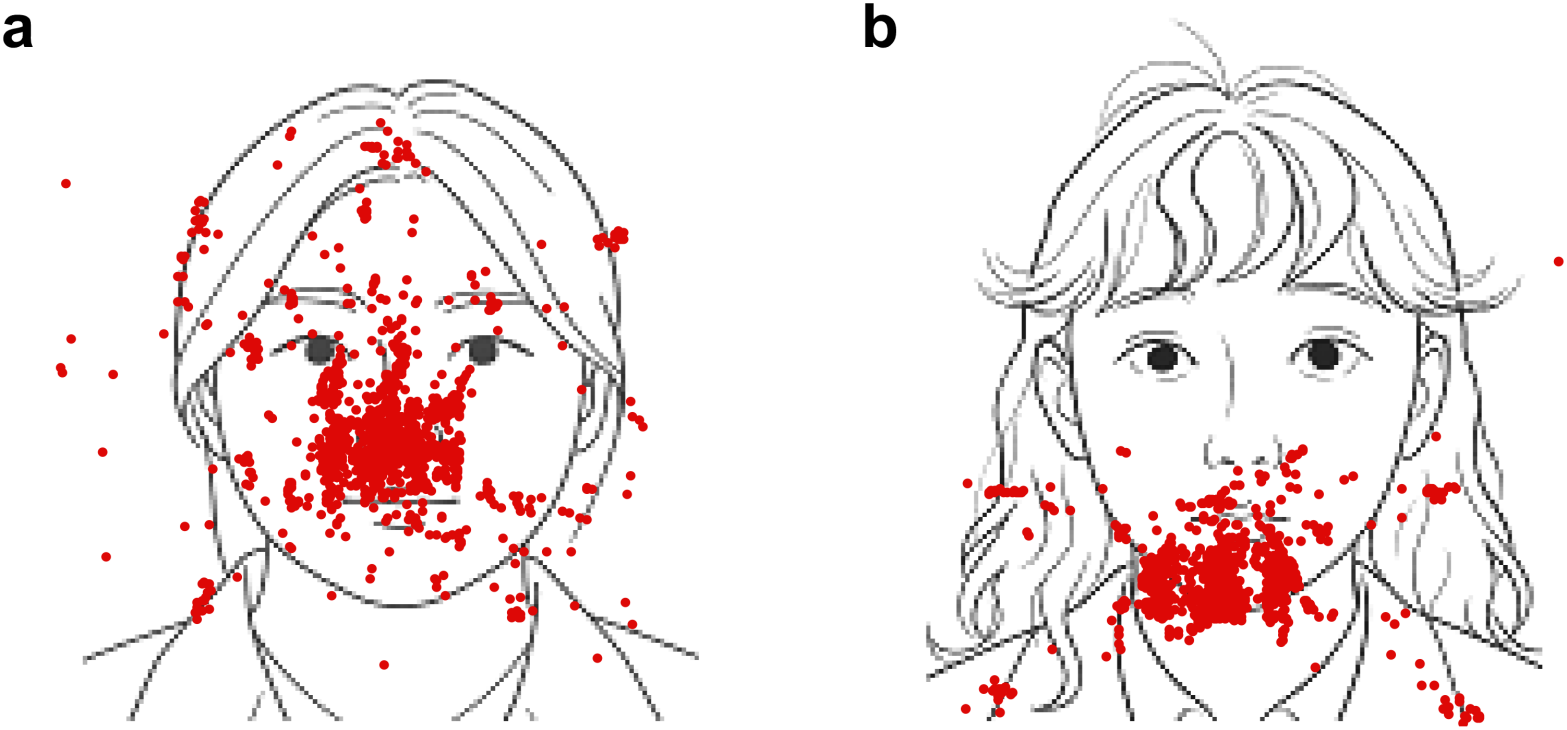
Representative examples of gaze pattern. The red dots represent gaze points in a single trial. **(a)** A case of a healthy individual when viewing their self-face. **(b)** A case of a patient when viewing their self-face. These line drawings were created using only the positional information of the participants’ facial features (hair line, eyebrows, eyes, nose, mouth, and ears) and contours.

**Figure 4:**
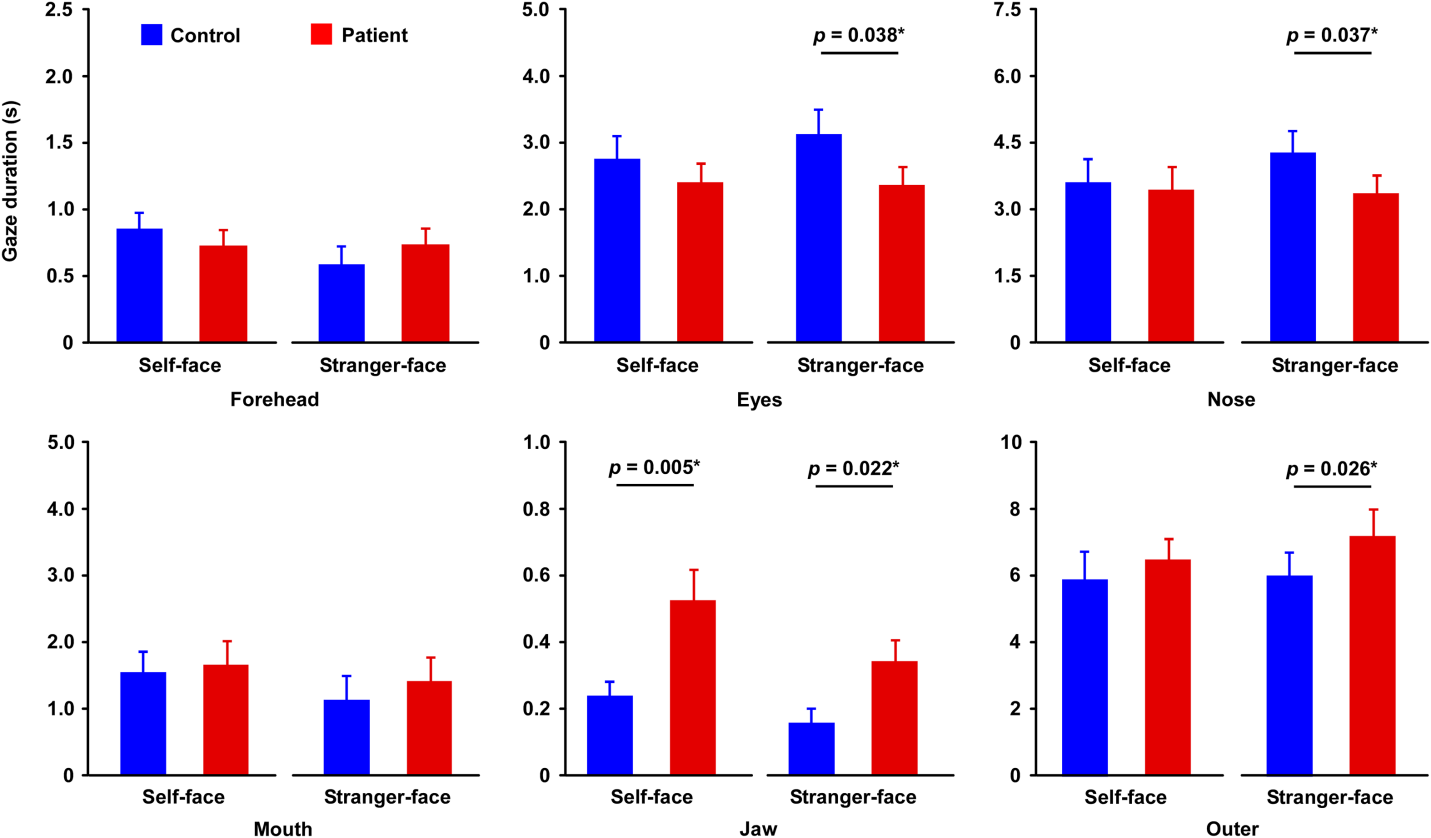
Results of gaze duration. Gaze duration across six facial regions for self- and stranger-face images. In the stranger-face, patients fixated significantly longer on the jaw and outer regions compared with controls, while they showed shorter gaze on the eyes and nose regions. In addition, patients fixated significantly longer on the jaw regions compared with controls, in the self-face. Asterisks indicate significant differences (*p < 0.05). Error bars represent SEM.

Overall, healthy individuals tended to gaze evenly across the face image (**Figure 3a**), whereas patients often showed concentrated gazes on the jaw region or directed their gaze toward outer regions of the face (**Figure 3b**). In the gaze duration (**Figure 4**), Mauchly’s test indicated that the assumption of sphericity was violated (region: *p* < 0.01; region x face identity: *p* < 0.01); therefore, Greenhouse–Geisser–corrected degrees of freedom were used. The three-way (region x face identity x group) ANOVA showed that there was significant difference in main effects of region (F_5, 490_ = 260.140, *p* < 0.0001, *η*^2^ = 0.728), while there were no difference in main effect of face identity (F_1, 98_ = 2.141, *p* = 0.147, *η*^2^ = 0.021) and of group (F_1, 98_ = 0.320, *p* = 0.573, *η*^2^ = 0.003). There were significant differences in the interactions of region and face identity (F_5, 490_ = 5.252, *p* = 0.003, *η*^2^ = 0.051), whereas the interactions of region and group (F_5, 490_ = 2.617, *p* = 0.059, *η*^2^ = 0.026) and of region, face identify, and group (F_5, 490_ = 2.461, *p* = 0.071, *η*^2^ = 0.024) did not reach statistical significance after the correction (**Supplementary Table 1**). There was no difference in the interaction of face identity and group. Exploratory *post hoc* test revealed that, compared to control, patients had long duration in the jaw region on self-face (*p* = 0.005) and stranger-face (*p* = 0.022) and in the outer region on stranger-face, while patients had short duration on in the eyes region on stranger-face (*p* = 0.038) and the nose region on stranger-face (*p* = 0.037).

### Relationship between BICI score and gaze duration

**Figure 5:**
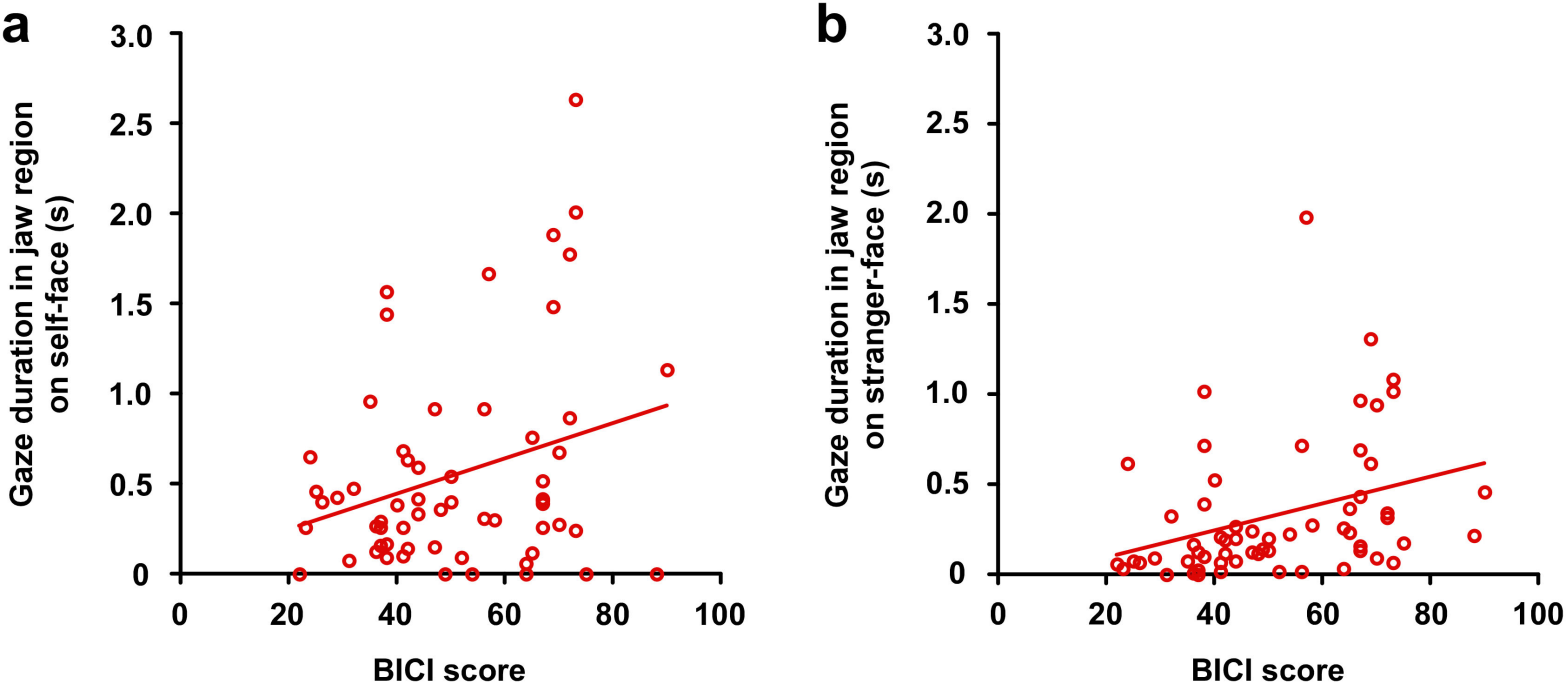
Associations between BICI scores and gaze duration in patients. **(a)** Jaw-region gaze on self-faces associated positively with BICI scores. **(b)** Jaw-region gaze on stranger-faces associated positively with BICI scores.

Although exploratory correlations suggested an association between jaw-directed attention and appearance-related concern, these findings should be interpreted with caution because the analyses were not corrected for multiple comparisons. In the patient group, the score of BICI associated with the gaze duration for the jaw (*r* = 0.383, *p* = 0.003, **Figure 5a**) and outer region (*r* = -0.376, *p* = 0.003) in the self-face, while the score did not associate with gaze duration in other regions of forehead, eyes, nose, and mouth (all *p* > 0.05) for the self-face. For the stranger’s face, the score associated with gaze duration in the mouth (*r* = 0.340, *p* = 0.008) and jaw regions (*r* = 0.387, *p* = 0.002) (**Figure 5b**), while the forehead, eyes, nose, and outer regions uncorrelated the score (all *p* > 0.300). These exploratory correlation analyses suggest that patients with higher BICI scores tended to spend more time looking at their jaws. In the control group, the score of BICI did not associate with gaze duration for the all regions of forehead, eyes, nose, mouth, jaw, and outer regions for the self-face (all *p* > 0.500). Similarly, for the stranger-face, the score did not associate with gaze duration for the all regions of forehead, eyes, nose, mouth, jaw, and outer regions (all *p* > 0.100).

## Discussion

The present study investigated the relationship between appearance-related psychological traits and gaze behavior in individuals with jaw deformities using eye-tracking. These findings indicate that patients showed region-specific differences in gaze patterns. This pattern of feature-focused attention is phenomenologically similar to attentional biases reported in previous eye-tracking studies of appearance-related concern, including those observed in BDD (4–6, 17). Such attentional biases have been reported across a range of populations with elevated appearance-related concern, suggesting that feature-focused visual attention may reflect general appearance-monitoring processes. The present findings extend this literature by demonstrating similar attentional patterns in individuals with objectively verifiable jaw deformities.

Increased attention to the jaw region was observed not only during self-face viewing but also when participants viewed others’ faces. At least two interpretations can account for the observed attentional pattern. First, increased gaze on the jaw region may reflect feature-based or salience-driven attentional processes (18), whereby visually prominent facial features preferentially capture attention during face perception (19). Second, the findings may reflect generalization of habitual feature monitoring, such that repeated attention toward a specific appearance-related feature results in a stable attentional tendency that extends across perceptual contexts. This account is consistent with models suggesting that attentional biases can become habitual and generalize beyond the original context in which they are formed (20–22).

A third possible interpretation concerns self–other representational overlap, which has been proposed in theoretical models of social cognition and self–other processing. According to this perspective, representations of the self and others may partially rely on shared cognitive or neural systems, allowing attentional biases initially related to self-processing to influence the perception of others (23). Prior eye-tracking studies have discussed similarities in attentional patterns during self-face and other-face viewing, raising the possibility of generalized self–other attentional patterns (4–6). However, the present data do not provide direct evidence in support of this interpretation. Accordingly, behavioral similarity should not be interpreted as evidence for shared representational mechanisms.

From a clinical standpoint, these findings suggest that distress among individuals with jaw deformities may reflect not only morphological characteristics but also perceptual-cognitive processes related to appearance monitoring. These processes are likely to represent adaptive or learned appearance-monitoring mechanisms associated with objectively verifiable facial differences in orthognathic patients. This has direct relevance for the preoperative and postoperative management of orthognathic and reconstructive patients (7, 11). Psychological screening for dysmorphic appearance concern could help identify individuals at risk of postoperative dissatisfaction despite objective improvement. This integrated framework resonates with the growing recognition of the need for multidisciplinary approaches in appearance-related conditions (2). Conceptualizing attentional bias as a modifiable cognitive mechanism allows clinicians to move beyond a purely morphological paradigm toward a biopsychosocial understanding of surgical outcomes. Such an approach may improve not only patient satisfaction but also long-term psychological well-being. Similar integrative strategies have been suggested for other disorders involving altered attentional bias processing, such as obsessive-compulsive and social anxiety disorders (24, 25).

Several limitations warrant consideration. First, because the present study employed a cross-sectional design, causal inference regarding the relationship between attentional bias and appearance-related concern is not possible. Nevertheless, recent experimental studies have demonstrated that manipulating visual attention toward or away from specific body or appearance features can influence body and appearance representations (26, 27). These experimental findings provide an important contextual background suggesting that attentional processes may play a causal role in shaping appearance-related representations. However, because attentional allocation was not experimentally manipulated in the present study, causal claims remain beyond its scope. Second, participants were Japanese adults recruited from a single institution, which may limit generalizability. Cultural norms regarding facial aesthetics and modesty could modulate the manifestation of dysmorphic concerns; thus, cross-cultural replication is essential (28, 29). An important limitation is that the present study focused on dysmorphic appearance concern as a dimensional psychological characteristic assessed using a validated self-report measure. Accordingly, the findings provide insight into psychological variation in appearance-related concern and its association with visual attention. Furthermore, appearance-related attentional bias has been observed across diverse clinical and non-clinical populations, supporting its relevance as a general perceptual-cognitive mechanism related to appearance monitoring.

In conclusion, the present study demonstrates that individuals with jaw deformities exhibit elevated appearance-related concern and appearance-related attentional bias toward jaw features. These findings provide behavioral evidence of appearance-related attentional processes in orthognathic patients and highlight the role of appearance-related concern in shaping visual attention.

## Data Availability

The datasets presented in this study can be found in online repositories. The names of the repository/repositories and accession number(s) can be found below: Mendeley Data, doi: 10.17632/5m696dygjz.1.
