# Correction: Appearance-related attentional bias is associated with dysmorphic appearance concern in individuals with jaw deformity: an eye-tracking study

**DOI:** 10.3389/fpsyt.2026.1870497

**Published:** 2026-05-19

**Authors:** Kiki Matsuzawa, Motoyasu Honma, Shugo Haga, Sumire Ogura, Yuri Masaoka, Masahiko Izumizaki, Haruhisa Nakano

**Affiliations:** 1 Department of Orthodontics, Showa Medical University School of Dentistry, Tokyo, Japan; 2 Department of Physiology, Showa Medical University School of Medicine, Tokyo, Japan

**Keywords:** attentional bias, body dysmorphic disorder, body image concern inventory, eye-tracking, jaw deformity

In the **Abstract**, the following mistakes were present: a missing past participle resulted in a structurally-incomplete opening sentence; the term “eye-while” was used in place of “eye-tracking”; an “attentional” feature was alluded to without specifying which one; “prolonged gaze” was mentioned without reference to a region, and “gaze” was used alone without any clarifying detail; and two sentences were cut off, one of which also had an unclosed parenthesis.

The **Abstract** has been corrected to read:

“**Introduction:** Appearance-related attentional bias has been observed in body dysmorphic disorder (BDD) and in individuals with elevated appearance concern; however, it remains unclear whether similar attentional biases are observed in individuals with objectively verifiable jaw deformities. Appearance-related attentional bias has been observed across clinical populations with elevated appearance concern, highlighting its relevance as a general perceptual-cognitive mechanism associated with appearance monitoring.

**Methods:** Sixty patients with jaw deformities and 40 age-matched controls completed the Body Image Concern Inventory (BICI), a validated measure of dysmorphic appearance concern, and underwent eye-tracking while viewing photographs of their own and others’ faces. Gaze duration was analyzed across six facial regions (forehead, eyes, nose, mouth, jaw, and others), and associations between gaze patterns and BICI scores were examined.

**Results:** Patients exhibited significantly higher BICI scores than controls. During both self- and other-face viewing, patients showed prolonged gaze duration toward the jaw region. Furthermore, exploratory correlation analyses showed that gaze duration was positively associated with BICI scores within the patient group during viewing of both self and others’ faces.

**Discussion:** Individuals with jaw deformities exhibit elevated dysmorphic appearance concern and increased attention to perceptually salient facial features. These findings characterize appearance-related attentional biases in individuals with jaw deformities and provide insight into perceptual processes associated with dysmorphic appearance concern.”

The original version of this article has been updated.

