# Supplementary material for: Appearance-related attentional bias is associated with dysmorphic appearance concern in individuals with jaw deformity: an eye-tracking study
Source: Front Psychiatry. 2026 Apr 23;17:1739028. doi: 10.3389/fpsyt.2026.1739028 (PMC13149380; doi:10.3389/fpsyt.2026.1739028)
Supplement: Supplementary file 1 [file DataSheet1.pdf]

Supplementary Information

Supplementary Table 1. ANOVA list for gaze duration.

Factor	df	MS	F	p	η^2
Region (A)	5, 490	933.632	260.140	< 0.001	0.728
Face Identity (B)	1, 98	0.043	2.141	0.147	0.021
Group (C)	1, 98	0.006	0.320	0.573	0.003
Interaction A x B	5, 490	4.603	5.252	< 0.001	0.051
Interaction A x C	5, 490	9.32	2.617	0.024	0.026
Interaction B x C	1, 98	0.006	0.320	0.573	0.003
Interaction A x B x C	5, 490	2.461	2.461	0.032	0.024
